# Analytical Methods for Os Isotope Ratios and Re-PGE Mass Fractions in Geological Samples

**DOI:** 10.3389/fchem.2020.615839

**Published:** 2021-02-17

**Authors:** Zhuyin Chu

**Affiliations:** ^1^ State Key Laboratory of Lithospheric Evolution, Institute of Geology and Geophysics, Chinese Academy of Sciences, Beijing, China; ^2^ Innovation Academy for Earth Science, Chinese Academy of Sciences, Beijing, China

**Keywords:** Re-Os, PGE, analytical method, NTIMS, ICP-MS, geoscience

## Abstract

The recent advances in analytical methods of Re-Os and PGE in geological materials including sample dissolution, chemical separation, mass spectrometric determinations, as well as the developments of matrix-matched reference materials for data quality control are thoroughly reviewed. Further, the *in-situ* measurement methods for Re-PGE mass fractions and ^187^Os/^188^Os ratios, as well as the measurement methods for stable isotope ratios of Re and PGE are also briefly reviewed. This review stands as a comprehensive reference for researchers to consider in the development of measurement methods for Re-PGE mass fractions and ^187^Os/^188^Os ratios in geological materials.

## Introduction

Rhenium, Os, and other PGE (here PGE mainly referring to Ir, Ru, Pt, and Pd; Rh is a mono-isotopic element and not reported in most studies that measure PGE abundances using isotope-dilution methodologies, thus not is focused on this review) are classified as highly siderophile and strongly chalcophile/sulphophile elements (e.g., Shirey and Walker, 1998; Barnes and Ripley, 2016). In addition, Rhenium, Os, and other PGE also show significant organo-phile properties (e.g., Stein and Hannah, 2015). Among these elements, ^187^Re is decayed to ^187^Os through β^−^ decay (λ = 1.666 × 10^−11^ a^−1^, t_1/2_ = 42.3 Ga; Smoliar et al., 1996) and ^190^Pt is decayed to ^186^Os through α decay (λ = 1.477 × 10^−12^ a^−1^, t_1/2_ = 469.3 Ga; Begemann et al., 2001).

The Re-Os and PGE systems have been widely applied in geosciences in the last decades due to their unique geochemical characteristics (e.g., Allègre and Luck, 1980; Morgan, 1986; Shirey and Walker, 1998; Stein and Hannah, 2015; Day, 2013; Day et al., 2016a; Barnes and Ripley, 2016; Harvey and Day, 2016). The application fields mainly include: 1) tracing planetary formation and evolution, particularly for tracing the late accretion history of Earth, Mar and Moon’s mantle (e.g., Day et al., 2007; Walker, 2009; Walker, 2015; Day et al., 2016a; Walker, 2016; Tait and Day, 2018); 2) tracing the evolution of Earth’s mantle, particularly for subcontinental lithospheric mantle (SCLM) dating (e.g., Walker et al., 1989; Shirey and Walker, 1998; Chu et al., 2009; Rudnick and Walker, 2009); 3) tracing the source of volcanic rock systems (e.g., Xu et al., 2007; Chu et al., 2013; Day, 2013; Chu et al., 2017); 4) dating metal sulfide ore deposits, particularly using molybdenite and low-level highly radiogenic (LLHR) sulfide Re-Os geochronology methods (e.g., Du et al., 1995; Stein et al., 2000; Stein, 2014; Stein and Hannah, 2015); 5) tracing the relative proportions of crust and mantle contributions to sedimentary (e.g., Stein and Hannah, 2015) or magmatic sulfide ore deposits (e.g., Shirey and Walker, 1998; Barnes and Ripley, 2016); 6) tracing the sulfide and/or PGE alloy segregation histories in magmatic systems (e.g., Barnes and Ripley, 2016); 7) age determinations of sedimentations such as in black shales (source rocks for oils) (Ravizza and Turekian, 1989; Creaser et al., 2002; Stein and Hannah, 2015) and even organic-rich carbonates (Rooney et al., 2014; Zhao et al., 2015); 8) investigation of global paleo-environments in earth’s history (e.g., Ravizza and Peucker-Ehrenbrink, 2003; Turgeon and Creaser, 2008; Sato et al., 2013; Stein and Hannah, 2015); 9) dating of petroleum system and fingerprinting hydrocarbon-source rock correlation (Selby and Creaser, 2005a; Selby et al., 2007; Finlay et al., 2011; Finlay et al., 2012; Lillis and Selby, 2013; Georgiev et al., 2016). In addition, although still controversial (e.g., Ireland et al., 2011; Day and O’Driscoll, 2019), the Pt-Os system has potential for tracing the core-mantle interaction (Walker et al., 1997).

Nevertheless, since the Re, Os, and other PGE are usually found in low abundance in geological samples (commonly in pg·g^−1^ to ng·g^−1^ levels) and are mainly hosted in trace phases such as sulfides, alloys, and other trace phases, rather than the major silicates (e.g., Lorand and Luguet, 2016), precise determination of Os isotope ratios and Re, PGE mass fractions in geological materials is difficult (Reisberg and Meisel, 2002; Meisel and Horan, 2016). The extremely low procedural blank for Re, Os, and other PGE is a prerequisite for the low uncertainty ^187^Os/^188^Os ratio and Re-PGE mass fraction measurement results. In recent decades, a lot of progress has been made in analytical methods for Re-Os and PGE in geological (and environmental) materials, including sample digestion, chemical separation, mass spectrometric determinations, as well as the development of matrix-matched reference materials for data quality control. Accordingly, recent advances in analytical methods (mainly for bulk analysis) for Re-Os and PGE in geological samples are briefly reviewed here.

## Sample Dissolution

For bulk Re-Os and PGE analysis of geological materials, the first step is to dissolve the Re-Os and PGE-bearing phases in samples with low procedural blanks. Currently, the sample digestion methods for Re-Os and PGE analysis mainly include: 1) NiS fire assay; 2) Low-temperature acid attack; 3) Alkali fusion, or acid dissolution combined with alkali fusion techniques; 4) Carius tube acid digestion; and 5) HPA (High Pressure Asher) acid digestion.

### NiS Fire Assay

The NiS fire assay method can digest large masses (could be >10 g) of samples for the Re-Os and PGE analysis, thus can reduce the “nugget effect” as Re-Os and PGE are usually hosted in heterogeneously distributed trace minerals such as sulfides and alloys (Ravizza and Pyle, 1997; Reisberg and Meisel, 2002). The drawback for the NiS fire assay method is the relatively high reagent blank. Carbonyl nickel or purified nickel (e.g., Sun and Sun, 2005) and sublimed S are preferred for fusion to achieve a lower blank level.

Ravizza and Pyle (1997) developed a NiS fire assay method for Os isotope and PGE analysis from the same sample digest with low procedural blanks. After filtration of the insoluble residue resulting from dissolution of the NiS bead, the filter paper containing the PGE-rich concentrate can be digested with an oxidant such as HNO_3_ to distill Os for isotopic analysis first, then the residue is processed for further analysis of other PGE (e.g., Hassler et al., 2000; Sun et al., 2009). Nevertheless, Re cannot be quantitatively recovered by the conventional NiS pre-concentration, thus Re is usually determined on a separate sample aliquot. Sun et al. (2009) developed an improved Fe-Ni sulfide fire assay method to allow for determinations of Re, PGE mass fractions and Os isotopic ratios from one sample digest.

Some researchers digested large masses (could be up to >50 g) of rock samples using the NiS fire assay method to obtain sufficient Os (30–200 ng) for ultra-high precision ^186^Os/^188^Os measurements by NTIMS (Negative Thermal Ionization Mass Spectrometry), and Os blanks are <1 pg per gram of sample fused (e.g., Brandon et al., 1998; Ireland et al., 2011; Day et al., 2017).

### Low-Temperature Acid Attack

Low-temperature acid dissolution method means using HF + HCl + ethanol or HF + HBr in a PFA beaker for the sample digestion (Walker, 1988; Birck et al., 1997; Reisberg and Meisel, 2002). The advantage of the method is that an extremely low procedural blank can be achieved (Os blank <0.05 pg) (Birck et al., 1997; Gannoun et al., 2007). The disadvantage of the method is that complete dissolution of Os-bearing refractory phases, particularly for peridotites, often cannot be achieved (e.g., Meisel et al., 2003a; Qi et al., 2011).

Gannoun et al. (2007) have compared the HF-HBr PFA beaker dissolution with the Carius tube acid digestion for Re-Os analysis of MORB (mid-ocean ridge basalts). Their results indicate that both techniques yield undistinguishable Os mass fractions and ^187^Os/^188^Os ratios for MORB samples. Nevertheless, this is not true for all basalts, in particular for the very fresh clinopyroxene rich ones (e.g., Ishikawa et al., 2014).

Qi et al. (2011) developed a PFA bomb HF dissolution method for PGE analysis. Their results show that when using the PFA bomb method, even with a dissolution temperature of 190°C, the ultramafic rocks cannot be effectively digested by HF-HCl but can be effectively dissolved by HF-HCl-HNO_3_. As oxidized HNO_3_ is used, the method can only be used for PGE analysis other than Os.

### Alkali Fusion, Acid Dissolution Combined with Alkali Fusion Techniques

Fusion with NaOH/Na_2_O_2_ has been used with success to dissolve refractory minerals such as spinel, chromite and PGE alloys for Re-Os-PGE analysis (Morgan and Walker, 1989; Sun et al., 2001; Reisberg and Meisel, 2002; Meisel et al., 2003a). This method is useful in particular for total digestion of meteorites (e.g., Morgan and Walker, 1989; Becker and Walker, 2003; Yokoyama et al., 2007; Yokoyama et al., 2010). The main drawbacks for the alkali fusion are that the reagent blanks are usually quite high, and that spike-sample equilibration sometimes cannot be completely achieved (e.g., Qi and Zhou, 2008). In addition, typically only 0.5 g sample can be digested.

Acid attack combined with Na_2_O_2_ fusion has been applied for effective sample digestion for Re-Os-PGE analysis. For example, Qi et al. (2004) reported a profound acid attack combined with a Na_2_O_2_ fusion method to lower the blank level for PGE analysis other than Os, including the following steps: first, HF, HNO_3,_ and HCl are used to decompose 10 g of the sample; subsequently the fluoride residue from the acid digestion is minimized using H_3_BO_3_ for complexation; finally, minimal sodium peroxide is used for fusion of mini-residues.

### Carius Tube Acid Digestion

Shirey and Walker (1995) first adopted the Carius tube method (Gordon, 1943; Wichers, et al., 1944; Gordon et al., 1944) for Re-Os isotopic analysis. The Carius tube method permits the dissolution of samples in oxidizing solutions (usually reverse aqua regia) at high temperatures without the loss of volatile OsO_4_. Typically, 2 g of silicate samples can be digested. For digestion of a sulfide such as pyrite, and a bitumen sample, addition of H_2_O_2_ is proven to be helpful (e.g., Qi et al., 2010). The Carius tube acid digestion method can achieve low procedural blanks for Re-Os and PGE. It is well-accepted that HNO_3_ is the main contributor of Os blank (Birck et al., 1997). It is widely reported that HNO_3_ can be pre-treated with the H_2_O_2_ purging (and N_2_ bubbling) to reduce its Os blank (e.g., Qi et al., 2010; Chu et al., 2013; Wang and Becker, 2014; Chu et al., 2015a; Yang et al., 2015; Day et al., 2016b; Day et al., 2017; Seo et al., 2018) (note: this technique originally from R. Creaser, University of Alberta, Canada). Usually, after two H_2_O_2_ purging purifications of HNO_3_, the procedural blanks of Os for the Carius tube method could be <0.3 pg.

KClO_3_/NaClO_3_-HCl has been used for digestion of Ir, Ru metal with the Carius tube method (e.g., Wichers, et al., 1944; Meisel et al., 2001; Savard et al., 2010; Chu et al., 2015a; Ren et al., 2016).

Meisel et al. (2003a) argued that for a serpentinized peridotite reference material (RM), UB-N, temperatures between 230 and 240°C, digested in Carius tubes were insufficient to completely digest its HSE-bearing spinels and HSE alloys; the highest yields of HSE were obtained at temperatures of ≥300°C in the HPA. Differently, Harvey et al. (2011) found little difference between HPA and Carius tube results for UB-N.

Becker et al. (2006) reported a modified method (c.f., Gordon et al., 1944) by putting the sealed Carius tube into a pressure steel jacket containing dry ice. The CO_2_ pressure that builds up inside the steel pressure vessel upon heating balances the internal pressure in the Carius tube, such that the digestion temperature could be as high as 345°C. For some mantle xenoliths, samples digested at 345°C give higher Os mass fractions and lower ^187^Os/^188^Os ratios compared to digestions at 220–230 °C. Similarly, Qi et al. (2007) put the Carius tubes in a steel pressure vessel containing water to prevent the explosion of the Carius tubes; 12 g silicate sample can be digested, and the digestion temperature can be set to 320°C thus allowing a more completed digestion of refractory minerals.

For silicate samples, some researchers used de-silicification with HF, before or after dissolution using aqua regia in a Carius tube (e.g., Meisel et al., 2003a; Qi and Zhou, 2008; Ishikawa et al., 2014; Li et al., 2015; Day et al., 2016b). Qi and Zhou (2008) reported that, for ultramafic reference material (RM) OKUM, about 4–15% of the PGE are in the silicate phase, which cannot be leached out by aqua regia even when digested at 300 °C with the modified Carius tube technique. Ishikawa et al. (2014) reported that: 1) improved results in RM TDB-1 for Re and Ru are obtained by additional treatment with hydrofluoric acid before or after Carius tube processing (c.f. Table 1); 2) for RM BIR-1, it is necessary to use HF desilicification to improve HSE recoveries, particularly Ru (c.f. Table 1). Li et al. (2015) reported that: 1) for RMs BHVO-2, TDB-1, and AGV-2 the HF desilicification increases the Re extraction efficiency (by 9–15%) and thus a small proportion of Re likely resides in silicate phases; 2) for RMs WGB-1 and WPR-1, Re extraction efficiencies obtained by the Carius tube digestion with and without HF desilicification are similar, indicating that Re in these rocks may dominantly reside in sulfides; 3) HF desilicification increases Os extraction efficiency in some RMs (e.g., BHVO-2 and AGV-2), suggesting that a portion of Os-rich trace phases may occur as inclusions in the silicate phases at ∼75 μm sizes. Differently, Day et al. (2016b) demonstrated that no systematic differences in HSE abundances are observed between data obtained by the Carius tube method with and without HF-desilicification for some intraplate basalts. Further, Day et al. (2016b) emphasized that: 1) if a Carius tube acid digestion to liberate Os, followed by HF-desilicification to obtain Re and Pt abundances, the measured Re/Os and Pt/Os may not correspond with measured ^187^Os/^188^Os and ^186^Os/^188^Os; 2) the sample solutions after the HF acid desilicification are much more complex with respect to potentially interfering elements, thus more complex column chemistry procedures are usually required.

**TABLE 1:** Ultramafic-mafic rock, shale and road dust reference materials for Re-Os and PGE.

RM	Source	Lithology	Digestion	Test size (g)	N	Re	Os	Ir	Ru	Pt	Pd	^187^Os/^188^Os ± 2SD	Data source
Ultramafic rocks													
WPR-1/WPR-1a	CANMET/CCRMP	Peridotite	CT	2	6	10.8 (1.8)	16.2 (1.1)	15.9 (0.9)	23.6 (0.6)	300 (3.7)	238 (5.1)	0.14467 ± 0.00018	Chu et al. (2015a)
			HPA	0.5	3	10.8 (0.3)	18.5 (0.5)	16.7 (0.8)	22.8 (1.0)	298 (5.5)	258 (2.1)		Meisel and Moser (2004a)
			CT/HPA	≥1	19	10.7 (2.3)	16.9 (5.1)					0.1449 ± 0.0017	Day et al. (2016b)
			CT-HF	≥1	4	10.6 (2.8)	16.4 (1.8)					0.14361 ± 0.0075	Day et al. (2016b)
			CT	0.5–0.7	5	11.2 (0.7)	15.2 (2.1)					0.14483 ± 0.0052	Li et al. (2015)
			HF-CT	∼0.5	7	11.0 (1.1)	15.7 (1.4)					0.14451 ± 0.00036	
UB-N^a^	SARM-CRPG/CRNS	Lherzolite	HPA	2	14	0.206 (2.4)	3.71 (7.1)	3.38 (6.4)	6.30 (4.6)	7.42 (4.0)	6.11 (2.9)	0.1278 ± 0.0002	Meisel et al. (2003a); Meisel and Moser (2004a)
			CT	1–2	5	0.197 (6.5)	3.42 (10)	3.56 (16)	7.09 (16)	7.56 (4.0)	5.66 (6.2)	0.1270 ± 0.0002	Li et al. (2014)
			CT	∼1	9	0.210 (8.6)	3.97 (9.5)	3.47 (6.6)	6.85 (5.8)	6.98 (5.0)	5.55 (3.1)	0.1273 ± 0.0017	Ishikawa et al. (2014)
			CT-HF	∼1	4	0.203 (1.0)	3.57 (7.8)	3.26 (8.9)	6.70 (10)	6.82 (3.7)	5.67 (3.0)	0.1272 ± 0.0010	
GP-13	Out of stock	Lherzolite	CT/HPA	0.5–2	9	0.286 (2.5)	3.36 (3.1)	3.32 (6.4)	6.92 (3.1)	6.89 (8.1)	5.46 (8.4)	0.12633 ± 0.00086	Day et al. (2012)
			CT	2	5	0.310 (3.6)	3.74 (3.3)	3.41 (5.6)	6.86 (1.5)	7.30 (6.3)	5.53 (3.6)	0.12645 ± 0.00027	Puchtel et al. (2008)
			HPA	2	7	0.320 (7.1)	4.06 (0.6)	3.33 (2.8)	6.25 (6.3)	6.69 (10)	5.68 (4.7)		Meisel and Moser (2004a)
JP-1	GSJ	Peridotite	CT	1–2	10	0.0244 (7.4)	3.16 (17)	2.83 (10)	5.64 (13)	4.80 (20)	1.50 (16)	0.1208 ± 0.0013	Ishikawa et al. (2014)
			CT-HF	∼1	5	0.0234 (7.3)	2.98 (22)	2.68 (16)	5.49 (15)	4.10 (17)	1.34 (10)	0.1208 ± 0.0017	
OKUM	IAGeo	Komatiite	NiS-FA	15			0.98 (34)	1.00 (12)	4.33 (12)	11.0 (5.3)	11.4 (7.0)		Savard et al. (2010)
			HPA	2		0.566 (6.3)	0.79	0.943 (3.9)	4.15 (2.0)	11.4 (1.9)	12.2 (6.0)		
			HPA	0.5–1	6	0.4 (21)	3.61 (97)	0.897 (8)	4.44 (2)	21.1 (46)	11.8 (4)	0.2704−0.5677	Wang and Becker (2014)
			CT-HF	2	12		0.66 (6.5)	1.06 (4.5)	4.89 (3.3)	12.9 (2.9)	12.2 (2.6)		Qi and Zhou (2008)
			HPA									0.27	Meisel et al. (2013)
HARZ01	IAGeo	Harzburgite	CT/HPA	0.2–1	11	0.061 (18)	4.07 (17)	3.52 (21)	6.3 (23)	6.8 (18)	5.5 (18)	0.1261 ± 0.0027	Day et al. (2016b)
			NiS-FA	15			4.97 (10)	4.04 (7.2)	8.69 (16)	8.20 (8.1)	6.16 (8.7)		Savard et al. (2010)
			HPA	2		0.084 (43)	3.29 (21)	2.32 (17)	5.41 (34)	8.72 (16)	7.25 (29)		
			CT	2	4	0.094 (19)	3.73 (7.1)	2.72 (9.8)	6.75 (22)	9.31 (21)	6.24 (15)	0.12569 ± 0.00036	this study
MUH-1	IAGeo	Harzburgite										0.127	Meisel et al. (2013)
Mafic rocks													
TDB-1	CANMET/CCRMP	Diabase	CT	2	4	1.03 (0.9)	0.139 (15)	0.093 (21)	0.226 (0.3)	5.21 (3.5)	20.9 (1.4)	0.7557–1.0589	Chu et al. (2015a)
			HPA	2	7	0.794 (3.0)	0.117 (10)	0.075 (13)	0.179 (2.9)	5.01 (3.6)	24.3 (7.8)		Meisel and Moser (2004a)
			CT	1–2	5	0.847 (3.2)	0.109 (13)	0.067 (12)	0.192 (8.2)	4.84 (6.2)	21.0 (10)	0.7542–1.0477	Li et al. (2014)
			CT	1–2	20	0.928 (7.9)	0.147 (26)	0.080 (29)	0.196 (14)	4.89 (15)	23.2 (11)	0.529–1.295	Ishikawa et al. (2014)
			CT-HF	1	8	1.05 (2.6)	0.145 (19)	0.080 (21)	0.285 (4.5)	5.00 (12)	23.4 (6.9)	0.747–1.225	
			HPA-HF	1	16	1.04 (2.0)	0.122 (22)	0.074 (27)	0.294 (11)	5.09 (11)	22.1 (5.7)	0.5909–1.1643	
WGB-1	CANMET/CCRMP	Gabbro	CT	2	6	1.17 (3.0)	0.832 (74)	0.196 (9.4)	0.164 (17)	3.95 (14)	10.6 (5.6)	0.14934–0.19146	Chu et al. (2015a)
			HPA	2	8	1.15 (6.0)	0.54 (14)	0.211 (13)	0.144 (16)	6.39 (56)	13.9 (19)		Meisel and Moser (2004a)
			CT	1–2	5	1.14 (3.4)	0.399 (9.7)	0.196 (5.8)	0.153 (18)	4.29 (12)	13.9 (14)	0.1792–0.1931	Li et al. (2014)
BIR-1/BIR-1a	USGS	Basalt	CT	2	4	0.661 (0.9)	0.385 (7.0)	0.136 (4.3)	0.292 (3.3)	5.01 (4.0)	5.48 (1.2)	0.13347 ± 0.00008	Chu et al. (2015a)
			HPA	2	5	0.634 (4.5)	0.374 (12)	0.149 (21)	0.278 (24)	4.30 (5.1)	6.11 (3.9)		Meisel and Moser (2004b)
			CT	1	8	0.675 (1.1)	0.355 (5.7)	0.125 (4.8)	0.255 (2.0)	4.23 (2.6)	5.56 (1.8)	0.13372 ± 0.00080	Ishikawa et al. (2014)
			CT-HF	1	9	0.684 (0.70)	0.345 (6.9)	0.143 (5.1)	0.519 (2.0)	4.37 (5.1)	5.84 (1.5)	0.1330 ± 0.0015	
			HPA-HF	0.5–1	4	0.678 (1.0)	0.381 (6.8)	0.143 (5.6)	0.531 (4.1)	4.25 (3.3)	5.85 (2.4)	0.1331 ± 0.0010	
			CT	0.2–1	43	0.689 (1.6)	0.349 (12)					0.1337 ± 0.0010	Zhang et al., 2017
			HF-CT	0.3–1	20	0.696 (1.2)	0.329 (14)					0.13372 ± 0.00063	
BHVO-1	USGS	Basalt	HPA	1	5	0.298 (2.5)	0.084 (26)	0.085 (21)	0.215 (45)	3.08 (32)	3.06 (2.4)		Meisel and Moser (2014b)
BHVO-2	USGS	Basalt	HPA	2	4	0.543 (4.1)	0.101 (25)	0.058 (25)	0.129 (43)	10.1 (49)	2.94 (1.4)		Meisel and Moser (2014b)
			CT	2–4	6	0.523 (2.8)	0.120 (9.7)	0.0795 (6.1)	0.138 (3.0)	8.64 (25)	2.69 (3.2)	0.1465 ± 0.0032	Chu et al. (2015a)
			CT	1–2	5	0.550 (6.1)	0.076 (15)	0.065 (9.5)	0.102 (8.0)	7.86 (49)	2.85 (4.1)	0.1540 ± 0.0022	Li et al. (2014)
BCR-2^b^	USGS	Basalt	CT	4	3	11.7 (0.9)	0.024 (0.04)	0.0055 (3.8)	0.033 (9.1)	0.850 (47)	0.451 (78)	28.57 ± 0.22	Chu et al. (2015a)
			CT	0.8	5	12.0 (1.9)	0.022 (4.0)					25.6 ± 6.1	Li et al. (2015)
			HF-CT	1	5	12.3 (2)	0.022 (2.9)					23.5 ± 5.2	
Shale													
SCo-1^c^	USGS	Shale	HPA	2–3	5	1.01 (6.2)	0.090 (13)	0.048 (22)	0.096 (22)	1.15 (74)	0.838 (5.7)	1.16−1.47	Meisel and Moser (2004b)
			CT/CT-HF	1–2	29	1.02 (1.7)	0.085 (7.1)	0.022 (29)	0.035 (28)	0.460 (10)	0.753 (6.5)	1.37 ± 0.20	Ishikawa et al. (2014)
			HPA	0.5	5		0.072 (1.5)					1.44 ± 0.10	Sen and Peucker-Ehrenbrink (2014)
SDo-1	Out of stock	Shale	HPA	1	1	93.1	1.16	0.077	0.216	2.15	2.42	4.12	Ishikawa et al. (2014)
			CT (CrO_3_-H_2_SO_4_)	0.5–1	12	76.3 (12)	0.632 (17)					7.84 ± 0.55	Du Vivier et al. (2014)
			HPA	1	5					1.44 (5.9)	2.69 (36)		Finlay et al. (2012)
			CT/CT-HF	1–2	6	75.8 (3.2)	0.068 (4.7)	0.060 (16)	0.118 (23)	1.42 (8.6)	2.30 (4.4)	7.64 ± 0.55	Ishikawa et al. (2014)
SBC-1	USGS	Black shale	CT	0.4–0.8	8	11.2 (1.1)	0.102 (4.0)					4.89 ± 0.84	Li and Yin (2019)
			CT (CrO_3_-H_2_SO_4_)	0.3–0.5	10	11.0 (1.4)	0.098 (2.9)					5.19 ± 0.40	
			CT (H_2_O_2_:HNO_3_ = 5:1)	0.2–0.5	10	11.0 (0.90)	0.096 (1.1)					5.29 ± 0.16	
SGR-1b	USGS	Oil shale	CT	0.1–0.4	5	34.7 (0.31)	0.444 (0.74)					1.7883 ± 0.0086	Yin et al. (2017)
			CT (CrO_3_-H_2_SO_4_)	0.2–0.4	4	33.7 (4.4)	0.447 (1.6)					1.7905 ± 0.0024	
			CT (H_2_O_2_:HNO_3_ = 5:1)	∼0.2	8	34.9 (0.56)	0.435 (1.3)					1.7917 ± 0.0052	
Road dust													
BCR-723	IRMM	Road dust	CT	0.3–0.4	10	6.51 (1.1)	0.37 (5.3)	0.22 (26)	1.10 (11)	79.8 (3.8)	4.57 (8.7)	0.537 ± 0.022	Zhao et al. (2014)

Relative standard deviations (1 RSD, %) are shown in parentheses for Re, Os, Ir, Ru, Pt and Pd mass fractions. N represents number of analyses; CT and PHA mean Carius tube or HPA digestion with reverse aqua regia respectively; CT/HPA-HF means CT or HPA acid digestion, followed by hydrofluoric acid-desilicification; HF-CT/HPA means HF-desilicification, followed by CT or HPA acid digestion.

^a^ UB-N is only prepared in demand (Meisel and Horan, 2016).

^b^ BCR-2 was likely contaminated by MoS_2_ during the powder preparation (Meisel and Horan, 2016)

^c^ SCo-1 out of stock, SCo-2 now available.

Qi et al. (2010) developed an improved Carius tube for determination of low levels of Re and Os in pyrites. The pyrites are predigested in an open Carius tube, the produced Os is trapped by a chilled HCl solution. The trapped Os is then put back to the Carius tube for the further complete digestion. The technique allows increasing the sample mass of pyrites to about 3 g.

For Re-Os isotopic analysis of quartz-bearing molybdenite, Lawley and Selby (2012) used concentrated HF to pre-dissolve quartz to isolate molybdenite at room temperature in a PFA beaker, prior to Carius tube digestion.

Selby and Creaser (2003) reported that, in case of Re-Os isotope analysis for organic-rich sedimentary rocks such as black shales, CrO_3_-H_2_SO_4_ (20% CrO_3_ in 2 mol/L H_2_SO_4_) is preferred for sample digestion in the Carius tube. Different from aqua regia, CrO_3_-H_2_SO_4_ can selectively dissolve the hydrogenous Re and Os without dissolving or significantly leaching clastic or detrital minerals (Selby and Creaser, 2003; Kendall et al., 2004). This is critical for Re–Os geochronology and Os isotope stratigraphy of organic-rich sedimentary rocks. With the CrO_3_-H_2_SO_4_ digestion method, black shale Re–Os geochronology with an age measurement uncertainty <1% (even 0.5%) can be achieved (e.g., Kendall et al., 2004; Selby and Creaser, 2005b; Kendall et al., 2006; Zhu et al., 2013). Differently, Yin et al. (2017) used HNO_3_-H_2_O_2_ for digestion of organic-rich sedimentary rock to reduce the leaching Os from crustal detrital minerals (c.f. Table 1).

### HPA Acid Digestion Method

The HPA (high pressure asher) digestion method was first used for Re-Os and PGE analysis of silicate samples by Meisel’s group from Montanuniversität Leoben, Austria (Meisel et al., 2001; Meisel et al., 2003a; Meisel et al., 2003b; Meisel and Moser, 2004a; Meisel and Moser, 2004b). The method uses a high pressure asher (HPA, Anton Paar, Graz) which employs reusable quartz glass containers to maintain a high temperature and high pressure under controlled conditions to allow a rapid and more complete sample digestion. Up to 3 g of silicate sample powder can be digested in reverse aqua regia in the HPA vessels at 300–320°C. The HPA digestion method has a lower PGE blank level compared to the Carius tube method, particularly Pt and Pd (e.g., Meisel et al., 2001; Meisel et al., 2003b). Thus, the HPA method is particularly useful for digestion of low PGE samples such as Apollo lunar basalts, which contain extremely low Re, Os and other PGE (at least twenty times lower than terrestrial basalts) (e.g., Day et al., 2007; Day and Walker, 2015). The main disadvantage of this technique is the high cost of the apparatus. Some researchers argued that even with the HPA method, for some silicate samples HF de-silicification is still necessary (e.g., Dale et al., 2012; Ishikawa et al., 2014).

Chen and Sharma (2009) and Seo et al. (2018) used HPA to allow for digestion of as much as 50 ml seawater or snow water, using 0.5 ml 8% CrO_3_-6 mol/L H_2_SO_4_ or 2.5 ml HNO_3_ + 1.5 ml H_2_O_2_ as oxidant, digested at 300°C to provide full Os oxidation.

Some researchers (e.g., Georgiev et al., 2016; Hurtig et al., 2020) used HPA for digestion of oil samples with HNO_3_ for Re-Os analysis, which essentially allows for larger samples to be processed compared to the Carius tube digestion.

## Chemical Separation

### Osmium Extraction

After sample digestion, two main classes of techniques can be used to separate Os from the dissolved sample matrix: distillation and liquid-liquid extraction (Reisberg and Meisel, 2002).

#### Distillation

After Carius or HPA digestion, the Os can be directly distilled (e.g., Shirey and Walker, 1995; Chen and Sharma, 2009). For alkali fusion or NiS fusion methods, the Os can be oxidized by the addition of a strong oxidizing agent, such as Ce(SO_4_)_2_ + H_2_SO_4_, CrO_3_ + H_2_SO_4_, HNO_3,_ or H_2_O_2_ solution (e.g., Walker, 1988; Morgan and Walker, 1989; Ravizza and Pyle, 1997; Markey et al., 1998; Hassler et al., 2000; Sun et al., 2001; Sun et al., 2009). Traditionally, the distillations are performed in assembled Pyrex glassware and large quantities (∼100 ml) of distillation solution are used (e.g., Shirey and Walker, 1995; Markey et al., 1998; Sun et al., 2001; Sun et al., 2009). The distillation apparatus can be pre-distilled with the pure reagents to reduce the blank level. Nägler and Frei (1997) first used commercially available PFA (Savillex) vials and tubing for the miniaturized distillation. Some authors developed *in situ* Caius tube distillation methods without the use of distillation flask to further reduce the Os blank (e.g., Qi et al., 2010). Jin et al. (2013) developed a device for a batch of simultaneous Carius tube *in situ* distillations, which can offer significantly higher sample throughput. Woodhouse et al. (1999) developed a method for the direct distillation of Os from large volumes of seawater (1–1.5 L, addition of H_2_O_2_ in H_2_SO_4_ to oxidize the spiked Os) for subsequent Os isotopic analysis.

#### Liquid-Liquid Extractions

CCl_4_/CHCl_3_ extraction is the most frequently used method for extraction Os from the sample matrix (Cohen and Waters, 1996; Shen et al., 1996). After the sample is digested by the Carius tube, the Os can be extracted from the reverse aqua regia or CrO_3_-H_2_SO_4_ solution directly into the CCl_4_ or CHCl_3_. Subsequently, the Os is back-extracted by HBr.

Another frequently used method involves extraction of OsO_4_ into liquid Br_2_ developed by Birck et al. (1997). For the HF-HBr sample dissolution method, CrO_3_ dissolved in HNO_3_ and liquid Br_2_ are used for oxidation of Os to OsO_4_ and Br_2_ extraction (Birck et al., 1997). For the Carius tube or HPA dissolution method, as Os has already be oxidized to OsO_4_, it is plausible to perform the Os extraction without the addition of CrO_3_ (e.g., Paul et al., 2009; Seo et al., 2018). For example, Paul et al. (2009) used the HPA method for digestion of 50 ml surface or subsurface waters, at high temperature (≥250°C), using 0.5 ml H_2_O_2_ and 0.5 ml H_2_SO_4_ as an oxidant to oxidize Os species. Subsequently, the OsO_4_ is directly extracted by addition of 2 ml liquid Br_2_. In order to compensate for the small ratio of Br_2_ to water (about 2:50), they increased the extraction time and perform the procedure twice.

The Br_2_ extraction technique allows extremely low Os blanks to be achieved (Birck et al., 1997), permitting the analysis of extremely small quantities of Os. Levasseur et al. (1998) developed a technique by adding a mixture of Br_2_, CrO_3,_ and H_2_SO_4_ to a spiked water sample (∼50 ml) in a 120 ml PFA pressure vessel, and heating at 90°C in an oven for at least 72 h to achieve spike-sample equilibration. The Os is oxidized to OsO_4_ and directly extracted from water into Br_2_ for subsequent mass spectrometric measurement. The total procedural blanks are <20 fg.

#### Micro-Distillation

For NTIMS Os isotope ratio measurements, the final Os purification is usually performed using a micro-distillation method developed by Birck et al. (1997). Birck et al. (1997) reported that the Os recovery during micro-distillation is about 70–90%. Recently, Nakanishi et al. (2019) further investigated the factors that affect the Os recovery during micro-distillation. They found that the most critical factor controlling the chemical yield of Os during micro-distillation is the extent of the dilution of the reductant (HBr) in the tip of the conical beaker by H_2_O evaporated from the oxidant. The H_2_O evaporation (and resultant HBr dilution) is suppressed by minimizing the volume of the oxidant solution and increasing the concentration of H_2_SO_4_ in the CrO_3_-H_2_SO_4_ oxidant solution as high as possible. In addition, the micro-distillation temperature should be <80°C, to reduce the evaporation of H_2_O into the HBr.

Day et al. (2016b, 2017) performed two or three micro-distillations to further improve the Os purity for NTIMS Os isotope ratio measurements.

Pearson et al. (1998); Harvey et al. (2006); Gannoun et al. (2007); Harvey et al. (2011) and Warren and Shirey (2012) used a method similar to the micro-distillation method for Os isotopic analysis of individual hand-picked sulfide grains. Rhenium and Os spikes and the sulfide grains are put onto the inverted cap of the conical 5 ml PFA vial and dried, CrO_3_-H_2_SO_4_ is then added to cover the sulfide grains to do the micro-digestion and micro-distillation. Pearson et al. (1998) have compared replicate micro-digestion dissolutions of single sulfide grains with a mini Carius-tube dissolution of a large, multi-crystal aggregate. The results indicate that the micro-digestion technique is effective at digesting the sulfides, and in achieving Os spike–sample equilibration.

### Separation of Re and the PGE

#### Separation of Re

In the case of only Re-Os analysis, the most commonly used method for Re separation is the anion exchange method (AG1-X8) (e.g., Morgan and Walker, 1989; Pearson and Woodland, 2000; Chu et al., 2015a). Re can be further purified by a small secondary anion-exchange column (e.g., Morgan and Walker, 1989; Du et al., 2004) or single anion beads clean-up method (Georgiev et al., 2018). As Cr^6+^ may be retained by the anion exchange resin as CrO_4_^2−^ to some extent, when CrO_3_-H_2_SO_4_ digestion method is used, it is necessary to reduce Cr^6+^ to Cr^3+^ using C_2_H_5_OH, H_2_O_2_, or H_2_SO_3_ (e.g., Selby and Creaser, 2003) before sample loading onto the column.

Another frequently used method to separate Re from sample matrix is NaOH (>5 mol/L)-acetone extraction (Du et al., 1995; Du et al., 2004; Georgiev et al., 2018). The NaOH can be pre-purified by acetone extraction to remove Re. The Re extracted by acetone can be further purified by a small anion exchange column (Du et al., 2004) or a resin bead clean-up step (Georgiev et al., 2018) if NTIMS method is applied for the Re measurement.

Sun et al. (2010) used only HNO_3_ for digestion of molybdenite in a Carius tube; after Os distillation, the Re is directly measured by ICP-MS without additional purification.

#### Separation of Re and Ir-Ru-Pt-Pd

When isotope-dilution (ID) method is applied, the most frequently used methods are cation (e.g., Meisel et al., 2003b; Qi et al., 2004; Shinotsuka and Suzuki, 2007; Fischer-Gödde et al., 2011; Li et al., 2014) or anion exchange methods (e.g., Rehkämper and Halliday, 1997; Pearson and Woodland, 2000; Chu et al., 2015a).

##### Cation Exchange

PGE and Re exist as chloro-complex anions and ReO_4_^−^ respectively in dilute HCl media, while major matrix elements exist as cations. Thus, PGE and Re can be separated collectively from sample matrix with high recovery using the cation exchange methods (e.g., Fischer-Gödde et al., 2011; Li et al., 2014). The main drawback with cation exchange separations is that relatively large amounts of resin are necessary to absorb the non-PGE ions to provide a good analyte/matrix separation and cleaning of the resin with large volumes of clean acid is required. In addition, some interfering elements such as Zr, Hf may not be efficiently separated from PGE (ZrO interferes with Pd; HfO interferes with Ir-Pt) (Qi et al., 2004; Shinotsuka and Suzuki, 2007; Li et al., 2014).

Meisel et al. (2003b) developed a low pressure (application of 2 Bar N_2_) cation exchange method with a long and thin column (1 m length, 4.2 mm inner diameter) that is coupled to a quadrupole ICP-MS for on-line matrix separation to have a full control over interference removal (e.g., Pd from Cd). Qi et al. (2004); Qi et al. (2007) used Te-coprecipitation to collect PGE from Na_2_O_2_ fusion or modified Carius tube digestion first, followed by a column packed with cation exchange (upper part) and HDEHP extraction (lower part) resins for further interfering elements removal. They concluded that the HDEHP resin is very efficient for removal of the Zr and Hf. Similarly, Ren et al. (2016) used a tandem assembly of mini-cation and Ln resin columns (both 5 mm inner diameter, 50 mm length) for the purification of Ru, Rh, Pd, Ir and Pt after a fire assay sample digestion. Shinotsuka and Suzuki (2007) further purified the PGE separated from cation exchange columns by solvent extraction using N-benzoyl-N-phenylhydroxylamine (BPHA) as an extractant to remove Zr, Hf, Mo. Differently, Li et al. (2014) coated the BPHA extractant onto an Amberchrom CG-71 m chromatographic grade material to make a column for a further removal of Zr, Hf and Mo from PGE. More recently, Zhou et al. (2019) reported that the abundance of interfering elements tends to increase in the eluent when conventional ion-exchange purification procedures are applied to de-silicified samples for Carius tube or HPA digestion method. They used an Ln column to further remove Zr, Hf after a cation exchange separation of PGE.

##### Anion Exchange

In contrast to cation exchange, with the anion exchange method, the PGE and Re are strongly retained on the resin (typically AG 1−X8) in dilute HCl media as chloro-complex anions and ReO_4_^−^ respectively, while most major and trace elements pass through the column directly (e.g., Rehkämper and Halliday, 1997; Pearson and Woodland, 2000; Meisel et al., 2001; Chu et al., 2015a). Thus, a small amount of resin is sufficient for the separation. The anion exchange method has the advantage of being able to separate Re and PGE into different fractions in subgroups such as Re + Ru and Pt + Ir, and thus is suitable for ID measurements of Re, Ru, Ir-Pt and Pd individually by MC-ICP-MS with high precision (e.g., Day et al., 2003; Chu et al., 2015a). The main drawback for anion exchange method mainly includes: 1) it is required to use high molarity acids (HCl or HNO_3_) to elute Re-PGE, particularly Ir-Pt and Pd; 2) the recovery of some elements such as Ir, Ru, Pd is relatively low and unstable (e.g., Chu et al., 2015a). Meisel et al. (2001) digested the resin absorbed Re-PGE in an HPA and the solution was measured with ICP-MS directly to overcome these problems. Again, it is necessary to reduce Cr^6+^ to Cr^3+^ with H_2_O_2_ or C_2_H_5_OH prior to anion exchange chromatography for high Cr sample (e.g., Meisel et al., 2003a; Dale et al., 2012). In addition, Zr and Hf can also form stable anion complexes in HCl media, which can sometimes be absorbed by the anion resin, creating oxide interferences on Pd (ZrO^+^) and Ir-Pt (HfO^+^) (Pearson and Woodland, 2000; Meisel et al., 2001; Chu et al., 2015a; Day et al., 2016b). Some researchers used 1 mol/L HCl-1 mol/L HF to elute Zr-Hf prior to PGE elution (Pearson and Woodland, 2000; Day et al., 2016b). Chu et al. (2015a) used an Ln column for secondary purifications of the Ir-Pt and Pd to remove Zr, Hf.

It should be noted that, although the PGE belong to the least abundant group of elements in the Earth’s crust, they may be elevated through anthropogenic influence on the environment, for example the use of automobile catalytic converters (Meisel et al., 2003b; Meisel and Horan, 2016). Consequently, as an example, the road dust has relatively high PGE content, particularly Pt, Pd (e.g., Zhao et al., 2014). Thus, a dust-free ultraclean lab is important for the PGE chemistry, particularly for low PGE samples.

## Mass Spectrometric Determination

### Os Isotopic Measurement

Currently, there are mainly two kinds of mass spectrometric method for Os isotopic analysis: NTIMS and MC-ICP-MS (Multiple Collector-Inductively Coupled Plasma-Mass Spectrometry). For samples with extremely high ^187^Os/^188^Os, such as molybdenite, quadrupole or single collector magnetic sector ICP-MS can also be applied.

#### NTIMS

The most widely used technique for Os isotopic analysis is NTIMS (Creaser et al., 1991; Völkening et al., 1991). Osmium is measured as OsO_3_^−^ on Pt-filament material using Ba(OH)_2_ as an electron emitter. The ion yield (ions detected by collector/atoms loaded) of OsO_3_^−^ can be >10% (Birck et al., 1997; Birck, 2001). Therefore, the NTIMS technique can analyze extremely low amounts of Os (down to several pico-grams) with high precision. For example, the NTIMS technique has been used for Os isotopic analysis of samples with extremely low Os mass fractions, such as seawaters (e.g., Levasseur et al., 1998; Woodhouse et al., 1999; Chen and Sharma, 2009), river and surface/subsurface waters (e.g., Sharma and Wasserburg, 1997; Paul et al., 2009), snow or ice water (Seo et al., 2018), ferromanganese crusts (e.g., Klemm et al., 2008), oils (e.g., Selby and Creaser, 2005a), hydrothermal sulfides (e.g., Stein et al., 2000), MORB (e.g., Gannoun et al., 2007) and lunar basalts (e.g., Day and Walker, 2015). For such kinds of studies, in addition to instrumentation, the most important factor that affects the data quality is the total procedural Os blank (should be extremely low).

Liu and Pearson (2014) and Wang et al. (2017) have used multi-collector Faraday cups equipped with 10^12^ and 10^13^ Ω amplifiers for high-precision Os isotopic analysis of low Os samples (could be as low as ∼0.025 ng), respectively.

An important problem associated with the NTIMS method is the correction of the minor oxide species such as Os^16^O_2_^17^O^−^ and Os^16^O_2_^18^O^−^ interferences on the signals of the primary Os^16^O_3_^−^ (e.g., Liu et al., 1998). Luguet et al. (2008); Chatterjee and Lassiter (2015) and Chu et al. (2015b) have reported in-run isobaric oxide correction methods for Os isotopic analysis with an improved precision.

For samples with low mass fractions of common Os and high mass fractions of radiogenic ^187^Os, such as molybdenite and LLHR sulfides, there is no isotopic ratios for internal mass fractionation correction. These problems can be resolved using a double Os spike (^188^Os–^190^Os, Markey et al., 2003; or ^186^Os-^188^Os, Qu et al., 2001) or using common Os as a double spike if the molybdenites contain no common Os (e.g., Suzuki et al., 1992; Selby and Creaser, 2001; Li et al., 2017). When utilizing NTIMS for Os measurements and a double spike or a common Os is used for mass fractionation correction, molybdenite Re-Os dating can provide ages with <0.1% level measurement uncertainties (Markey et al., 2003; Li et al., 2017).

#### MC-ICP-MS

Another technique used for Os isotopic analysis is ICP-MS (for isotopic analysis, mainly use MC-ICP-MS) (e.g., Schoenberg et al., 2000; Nowell et al., 2008a). The main advantage of ICP-MS measurements is the rapidity relative to NTIMS measurements. Nevertheless, currently, the Os ion yield, and thus, the precision of ICP-MS analysis is significantly lower than that of NTIMS [ion yield: traditional MC-ICP-MS, ∼0.08% for Os introduction in a reduced state (e.g., Nowell et al., 2008a); NTIMS >10% (e.g., Birck et al., 1997; Birck, 2001)]. Although the oxidized state of the Os (as OsO_4_ in H_2_O) for introduction into the ICP source has a much higher ion yield (at least 30 times higher than introduction of Os in a reduced state; e.g., Sun et al., 2001), the OsO_4_ has an extremely strong memory effect in the introduction system of ICP-MS (e.g., Sun et al., 2001; Sun et al., 2009). It has been reported that dilute NH_2_OH·HCl or H_2_NNH_2_·H_2_O can allow a more effective rinsing between Os measurements for the OsO_4_ introduction method (Meisel et al., 2001; Sun et al., 2001; Sun et al., 2009). In addition, more complex interfering isobaric elemental ions are usually generated by the plasma, compared to the NTIMS method.

One advantage for ICP-MS is a rapid sparging technique that can be used to extract OsO_4_ from the sample solution and transferred directly into the machine for isotope ratio measurements (Hassler et al., 2000; Norman et al., 2002; Nozaki et al., 2012; Sen and Peucker-Ehrenbrink, 2014). The elimination of the nebulizer and spray chamber assembly with the sparging method minimizes memory problems. A problem is that signal intensities usually decrease throughout a sparging analysis, which limits the analytical precision of the Os isotope ratio measurement results. Jin et al. (2011) developed a method to directly transfer OsO_4_ vapor from a Carius tube into an ICP-MS source for Os measurement, with a solution containing Ir being aspirated for mass bias correction.

Nozaki et al. (2012) reported that the sparging method combined with CrO_3_-H_2_SO_4_ digestion of organic-rich sedimentary rocks and multi-ion-counters (MIC) in the mass spectrometry is expected to be a powerful tool for reconstructing the secular change in marine Os isotope compositions with high sample throughput. Sen and Peucker-Ehrenbrink (2014) suggested to use the fast-sparging method to screen large numbers of oil and their source rock samples for finding a set of samples with spread in ^187^Re/^188^Os for further high-precision isochron analyses by N-TIMS.

### Determination of Re and PGE Mass Fractions

Rhenium and the PGE mass fractions are usually measured by ICP-MS with ID method. Some labs use NTIMS for determination of Re (e.g., Markey et al., 2007; Liu and Selby, 2018). When NTIMS is used, it should be noted that trace amount of Re is probably present in the Pt filament (e.g., Day et al., 2003), and thus often Ni filament is applied with Ba(NO_3_)_2_ as an ion emitter. In addition, the thermal fractionation effect for Re isotopes with the NTIMS method is difficult to correct. Suzuki et al. (2004) developed a method for low uncertainty Re isotope ratio measurements by NTIMS using a total evaporation technique.

Measurements of isotope ratio of the Re and PGE for ID quantification is by ICP-MS, either single collector analysis using a magnetic sector field (ICP-SFMS, e.g., Element XR) (e.g., Fischer-Gödde et al., 2011; Wang and Becker, 2014) or quadrupole (ICP-QMS) mass spectrometer (e.g., Pearson and Woodland, 2000; Meisel et al., 2003b; Qi et al., 2004; Li et al., 2014; Day et al., 2016b), or by MC-ICP-MS (e.g., Day et al., 2003; Chu et al., 2015a). The mass bias effects are usually corrected by the sample-standard bracketing (SSB) method (e.g., Day et al., 2003; Chu et al., 2015a). In case of only Re analysis, the mass bias effect can also be corrected using doped Ir (e.g., Schoenberg et al., 2000; Day et al., 2003). The ICP-QMS and ICP-SFMS can measure all of the PGE and Re in a single aliquot and monitor the interfering elements in-run. Comparatively, the MC-ICP-MS technique has the highest sensitivity (or signal/noise ratio), thus is more suitable for analysis of extremely low amounts of Re and PGE (e.g., Day et al., 2003). However, it is difficult to monitor all interfering elements in run with the MC-ICP-MS technique. It is useful to use a desolvator to reduce isobaric oxide interferences for PGE determination using ICP-MS (e.g., Pearson and Woodland, 2000; Fischer-Gödde et al., 2011).

## Stable Isotope Ratio Measurements for Re and PGE

Due to the unique chemical characteristics of Re and PGE, the stable isotopic ratios of Re and PGE are also useful for investigating the planetary formation, differentiation processes, as well as the Earth’s paleoredox history (Hoefs, 2018), and particularly nucleosynthetic isotope variations in the solar-system (e.g., Becker and Walker, 2003; Yokoyama et al., 2007, 2010; Chen et al., 2010; Fischer- Gödde et al., 2015; Yokoyama and Walker, 2016).

Nanne et al. (2017) described high-precision MC-ICP-MS and N-TIMS Os isotope ratio measurement methods using a ^188^Os-^190^Os double spike technique. More recently, Zhu et al. (2018) provided a new mass fractionation correction method for MC-ICP-MS to determine the absolute Os isotope ratios which does not rely on Nier’s values (^192^Os/^188^Os = 3.083, Nier, 1937). They determined Os isotope ratios using NRC (National Research Council Canada) IRIS-1 Ir for normalization. The Ir isotope ratio of IRIS-1 has been certified by NRC, determining by MC-ICP-MS using NIST SRM 997 Tl and NIST SRM 989 Re for normalization (Zhu et al., 2017). It should be noted that, the Os isotope ratios traceable to NRC IRIS-1 Ir probably are closer to the “true values” but with a larger uncertainty compared to those arbitrarily normalized to ^192^Os/^188^Os = 3.083. A precise MC-ICP-MS technique to measure Re isotope variations has been presented by Miller et al. (2009). Yokoyama et al. (2007); Yokoyama et al. (2010) determined Os isotope ratios in chondrites in order to resolve possible nucleosynthetic isotope anomalies.

Creech et al. (2013); Creech et al. (2014) described a precise MC-ICPMS technique measuring ^198^Pt/^194^Pt ratios (using a ^196^Pt–^198^Pt double spike) relative to the IRMM 010 Standard. 11 international geological standard reference materials were determined for ^198^Pt/^194^Pt variations. Creech et al. (2017) described a MC-ICP-MS technique by measuring ^106^Pd/^105^Pd ratios (using a ^106^Pd–^110^Pd double spike) in a variety of terrestrial and extraterrestrial materials. Hunt et al. (2018) developed a method for measuring Pt isotope ratios of ion meteorite samples. More recently, Creech et al. (2020) used a MC-ICP-MS equipped with 10^13^ Ω amplifiers for Pt isotope ratio measurements of samples with low Pt abundances.

Ruthenium isotope ratios can be measured using either NTIMS (e.g., Becker and Walker, 2003; Chen et al., 2010) or MC-ICP-MS (Becker et al., 2002; Fischer- Gödde et al., 2015). Chen et al. (2010) used a mixture of Ba(NO_3_)_2_ and Ba(OH)_2_ (in the approximate ratio of 1:10) as ion emitter to improve the ionization efficiencies of RuO_3_^−^ for NTIMS analysis. The Ru can be separated from sample matrix by cation (Becker et al., 2002; Becker and Walker, 2003; Fischer- Gödde et al., 2015) or anion exchange (Chen et al., 2010) chromatography and then be further purified by distillation or micro-distillation methods (distillation as RuO_4_) similar to that for Os. The above techniques have been used to determine Ru isotope ratios of meteorites to resolve Ru isotope anomalies (e.g., Becker and Walker, 2003; Chen et al., 2010; Fischer- Gödde et al., 2015). Recently, Hopp et al. (2016) developed a MC-ICP-MS method to measure precise ^102^Ru/^99^Ru ratios (using a ^98^Ru–^101^Ru double spike) to resolve natural Ru stable isotope variations.

## *In-situ* Techniques for Os Isotope Ratio and Re, PGE Mass Fraction Measurements

### LA-(MC)-ICP-MS

LA (Laser ablation)-MC-ICP-MS is the most important *in-situ* technique for Os isotope ratio measurements. A problem associated with LA-MC-ICP-MS method is the isobaric interference of ^187^Re on ^187^Os (Nowell et al., 2008b). Thus, the method is only suitable for ^187^Os/^188^Os measurements of minerals with high Os but low Re mass fractions (at least ^187^Re/^188^Os ratios <0.5, Nowell et al., 2008b), such as primary mantle sulfides (e.g., Pearson et al., 2002; Alard et al., 2002; Alard et al., 2005) and Os-bearing alloys (e.g., Hirata et al., 1998; Walker et al., 2005; Nowell et al., 2008b). Another problem is the ^186^W interference on ^186^Os if ^186^Os/^188^Os is required to measure (Hirata et al., 1998; Walker et al., 2005; Nowell et al., 2008b). For Pt-bearing PGA (Platinum Group Alloy) analyses by LA-MC-ICP-MS, it is the better to use ^189^Os/^188^Os rather than ^192^Os/^188^Os for instrumental mass fractionation correction because of isobaric interferences on ^192^Os from ^192^Pt (Hirata et al., 1998; Nowell et al., 2008b).

Pearson et al. (2002) firstly established a LA-MC-ICP-MS technique for direct measurement of mantle sulfides containing Os at trace element (tens or hundreds of μg·g^−1^) levels. For sulfides enclosed in silicate minerals (e.g., olivine) larger than 50 μm in diameter and with Os contents >40 μg·g^−1^, the method can give ^187^Os/^188^Os ratios with a precision of 0.1% (2 RSE). The technique has been used to demonstrate distinct differences in Os isotopic composition between sulfide enclosed in silicates and intrastitial sulfides in peridotites (Pearson et al., 2002; Alard et al., 2002; Alard et al., 2005). The sulfide inclusions in silicates preserve significantly lower ^187^Os/^188^Os than interstitial sulfides and accordingly produce significantly older and more realistic Re-Os age information. Interstitial sulfides typically have lower Os (10–30 μg·g^−1^), higher Re and give analyses with higher uncertainty (∼1–2%) but still provide valuable information.

Hirata et al. (1998) firstly used LA-MC-ICP-MS for Os isotope ratio measurements of PGE alloys such as iridosmines. Walker et al. (2005) used LA-MC-ICP-MS for ^186^Os/^188^Os and ^187^Os/^188^Os measurements of Os–Ir–Ru alloy grains from southwestern Oregon, United States. Compared to the SIMS (Secondary Ionization Mass Spectrometry) technique (e.g., Meibom et al., 2002; Meibom and Frei, 2002), the LA-MC-ICP-MS can measure isotope ratios of PGE alloys with a lower measurement uncertainty at a lower analytical cost. Nowell et al. (2008b) presented a rapid (40 s acquisition time) LA-MC-ICP-MS methodology suitable for applying Pt–Os and Re–Os geochronology approaches to single PGA grains, for use in dating chromitite deposits and identifying and dating multiple sources in alluvial PGA deposits.

The LA-ICP-MS techniques has been widely used for determination of PGE mass fractions in mantle sulfide (e.g., Alard et al., 2000; Lorand et al., 2008; Lorand et al., 2010; Lorand and Luguet, 2016). For the measurement, a synthetic PGE doped NiS bead (PGE-A) is used as an external calibration standard. Their results show that sulfides enclosed in silicate phases have high osmium and iridium abundances but low Pd/Ir ratios, whereas pentlandite-dominated interstitial sulfides show low osmium and iridium abundances and high Pd/Ir ratios. They interpreted the silicate-enclosed sulfides as the residues of melting processes and interstitial sulfides as the crystallization products of sulfide-bearing (metasomatic) fluids. Lorand et al. (2008); Lorand et al. (2010) used time resolved analysis by LA-ICP-MS to find the PGE alloy micro-nugget in mantle sulfide grains.

LA-ICP-MS has also been extensively applied for the PGE analysis of iron meteorites, chondrites, as well as some achondrite meteorite groups (Campbell and Humayun, 1999; Day et al., 2016a).

Parent-daughter (^187^Re–^187^Os) decoupling within molybdenite crystals has ever been confirmed by LA-(MC)-ICP-MS studies (Stein et al., 2003; Selby and Creaser, 2004).

### Scanning Electron Microscope, Synchrotron Radiation X-Ray Fluorescence and Atom Probe Microscopy *In-Situ* Techniques

The scanning electron microscope (SEM) technique has frequently been used to find and detect PGE micro-nuggets in rock samples (e.g., Luguet et al., 2007; Lorand et al., 2008; Lorand et al., 2010; Lorand and Luguet, 2016).

Kogiso et al. (2008) employed microbeam SR-XRF to detect micrometer-scale platinum-group minerals in an orogenic lherzolite sample. Daly et al. (2017a) used SR-XRF for *in-situ* analysis of PGE-rich refractory metal nuggets in carbonaceous chondrites.

Atom probe microscopy (APM) is a relatively new *in situ* tool for quantifying elemental and isotopic compositions at a sub-nanometer spatial resolution (<0.02 μm^3^ in volume) in geological materials (Reddy et al., 2020). Parman et al. (2015) applied the laser-assisted APM technique for measurements of Pt, Fe, Ir, Ni, Rh, Ru, and Cu mass fractions and Pt, Ru, Ir isotope ratios of Pt_3_Fe (isoferroplatinum) inclusions in an Os-Ir alloy (osmiridium) grain. Daly et al. (2017b) performed APM analyses of sub-micrometer PGE-rich refractory metal nuggets contained within a Sc-Zr–rich ultrarefractory inclusion from the ALH 77307 CO3.0 meteorite. More recently, Daly et al. (2018) employed the laser-assisted APM technique to measure Re and Os isotope ratios of pure Os, pure Re, and synthetic Re-Os-bearing alloys, demonstrating that the APM technique permits nanoscale Os isotope ratio measurements of Os-bearing alloys to yield similar, though less precise, ages to NTIMS analyses. At current, development and application of the analytically expensive and highly-specific APM technique in geosciences is still in its early stages (Reddy et al., 2020).

## Reference Materials for Data Quality Control of Re-Os and PGE Analysis

Reference materials (RMs) play a key role in method development and quality control in routine analyses (Meisel and Horan, 2016). Table 1 lists recently published ^187^Os/^188^Os and Re-PGE mass fraction data for the frequently used rock RMs (except for BCR-723, which is an environmental RM).

### Ultramafic Rock Reference Materials

Among ultramafic RMs (Table 1), WPR-1/WPR-1a (altered peridotite), UB-N (serpentinized Lherzolite), and GP-13 (Lherzolite) are relatively easy to digest and proven to be relatively homogenous in Re-PGE mass fractions and ^187^Os/^188^Os ratios at 0.5–2 g sample size levels (Table 1) (e.g., Meisel et al., 2003a; Meisel and Moser, 2004a; Putchtel et al., 2008; Li et al., 2014; Day et al., 2012; Chu et al., 2015a; Day et al., 2016b), indicating that the Re-PGE are mainly hosted in base metal sulfides (e.g., Meisel et al., 2003b; Meisel et al., 2013; Day et al., 2016b; Meisel and Horan, 2016). Nevertheless, UB-N is only prepared on demand (Meisel and Horan, 2016); this means that the PGE composition can possibly change from batch to batch. GP-13 is now not available.

MUH-1 (harzbugite) and OKUM (komatiite) were prepared, bottled at the same time and tested for homogeneity. In addition their major and trace element mass fractions have been certified according to ISO Guides (Meisel and Horan, 2016). Qi and Zhou (2008) showed that OKUM is relatively homogeneous in PGE mass fractions at 2 g test portions (OPY-1 in Qi and Zhou (2008) is the same as OKUM; Meisel, personal communication) (Table 1). Nevertheless, Wang and Becker (2014) demonstrated that this RM is highly heterogeneous both in Re-PGE mass fractions and ^187^Os/^188^Os ratios, in particular between bottles. HARZ01 was also prepared by IAGeo limited as a harzbugite RM. It has been shown that harzbugite RMs (HARZ-01/MUH-1) are heterogeneous in Re-PGE mass fractions but relatively homogeneous in ^187^Os/^188^Os ratios (Table 1; Savard et al., 2010; Meisel et al., 2013; Day et al., 2016b; Chu, unpublished data). It is likely that the Re-PGE bearing phases in these harzburgite RMs (HARZ-01/MUH-1) are typically alloys and laurites (Meisel et al., 2013; Day et al., 2016b; Meisel and Horan, 2016), which are difficult to digest.

### Mafic Rock Reference Materials

BIR-1a has been proven to be homogenous both in Re-PGE mass fractions and ^187^Os/^188^Os ratios at 0.5–2 g test portions (BIR-1 is out of stock, now BIR-1a available) (Table 1) (e.g., Meisel and Moser, 2004b; Li et al., 2014; Ishikawa et al., 2014; Chu et al., 2015a; Zhang et al., 2017) and thus is recommended as a basaltic RM for Re-Os and PGE. Basaltic RM USGS BHVO-2 has been widely reported for Re-PGE mass fractions and ^187^Os/^188^Os ratios but not definitely homogeneous at a 2 g sample size level (Table 1) (e.g., Meisel and Moser, 2004b; Ishikawa et al., 2014; Li et al., 2014; Li et al., 2015; Chu et al., 2015a). USGS BCR-2 was found to have high Re (∼11.7 ng·g^−1^), low Os mass fractions (∼24 pg·g^−1^) and a very high ^187^Os/^188^Os (∼28.57) and seems homogenous at a 2 g sample size level (Table 1) (e.g., Chu et al., 2015a; Li et al., 2015). Nevertheless, BCR-2 was likely contaminated by MoS_2_ during the powder preparation (Meisel and Horan, 2016), thus cannot be regarded as a matrix-matched basaltic RM for Re-Os.

TDB-1 (diabase) and WGB-1 (gabbro) have been certified for Pd, Pt, and Au mass fractions by CCRMP, and widely used as mafic rock RMs for Re-Os and PGE analysis (Savard et al., 2010; Meisel and Horan, 2016). However, these RMs are not homogeneous in term of Re-PGE mass fractions and ^187^Os/^188^Os ratios at a 2 g test portion level (Table 1). The replicate analyses of Re-Os for TDB-1 and WGB-1 even show strong ^187^Os/^188^Os-^187^Re/^188^Os correlations to construct errorchrons (e.g., Ishikawa et al., 2014; Li et al., 2014; Chu et al., 2015a).

### Shale

USGS RMs SDo-1 and SCo-1 have been reported for Re-PGE mass fractions and ^187^Os/^188^Os ratios (e.g., Meisel and Moser, 2004b; Finlay et al., 2012; Du Vivier et al., 2014; Ishikawa et al., 2014; Sen and Peucker-Ehrenbrink, 2014; Table 1). Now SCo-1 is out of stock, but SCo-2 is available. SDo-1 is out of stock.

USGS RMs SBC-1 and SGR-1b are relatively homogeneous in Re, Os mass fractions and ^187^Os/^188^Os ratios in 0.2–0.5 test portions (Table 1) and thus recommended as Re-Os RMs for black shale (Li and Yin, 2019) and oil shale (Yin et al., 2017), respectively. Nevertheless, PGE mass fraction data have not reported for these RMs up to now.

### Road Dust

BCR-723 has been certified for Pt, Pd and Rh as a road dust RM by IRMM (Zischka et al., 2002). This RM has been recently reported for Re, Os, Ir, Ru, Rh, Pt and Pd fractions and ^187^Os/^188^Os ratios (Table 1) (Meisel et al., 2003b; Zhao et al., 2014).

### Molybdenite

JDC-1 and HLP-1 were launched as molybdenite Re, Os concentrations and Re-Os age RMs as described in detail by Du et al. (2004). Markey et al. (2007) developed a molybdenite Re-Os age RM, NIST RM 8599. They emphasized that, RM8599 is not a RM for Re and ^187^Os mass fractions, but only for the accuracy of ^187^Re–^187^Os ages. The Re and ^187^Os mass fractions vary but are perfectly coupled such that the age is highly reproducible. The reported Re-Os data for these molybdenite RMs are given in Table 2.

**TABLE 2:** Certified values for molybdenite reference materials (95% confidence).

RM	Re (μg·g^−1^)	Os (ng·g^−1^)	Age (Ma)	Reference
JDC-1	17.39 ± 0.32	25.46 ± 0.60	139.6 ± 3.8	Du et al. (2004)
HLP-1	283.8 ± 6.2	659 ± 14	221.4 ± 5.6	
NIST RM 8599			27.656 ± 0.022	Markey et al. (2007)

### Oil

NIST RM 8505 whole oil and its homogenized powered asphaltene separates were recommended as Re-Os matrix-matched oil RMs by Liu and Selby (2018). Hurtig et al. (2020) also reported Re-Os data for NIST RM 8505 crude oil and another oil RM, NIST RM 1634c. Although the Re, Os mass fractions for NIST RM 8505 whole oil reported by Hurtig et al. (2020) are consistently higher than those reported by Liu and Selby (2018), the ^187^Os/^188^Os data between the two labs are consistent within stated precisions. The reported Re-Os data for these oil RMs are summarized in Table 3.

**TABLE 3:** Reported Re, Os mass fraction and ^187^Os/^188^Os data for crude oil reference materials.

RM	Re (ng.g^−1^)	Os (pg.g^−1^)	^187^Os/^188^Os	Reference
NIST RM 8505 crude oil (mean ± 2SD, n = 20)	1.98 ± 0.07	25.0 ± 1.1	1.51 ± 0.01	Liu and Selby (2018)
Asphaltene separates from NIST RM 8505 crude oil (mean ± 2SD, n = 24)	16.52 ± 0.10	166.0 ± 0.9	1.64 ± 0.01	
NIST RM 8505 crude oil (mean ± 2SD, n = 8)	2.40 ± 0.27	30.1 ± 1.5	1.504 ± 0.050	Hurtig et al. (2020)
NIST RM 1634c (mean ± 2 SD, n = 3)	1.73 ± 0.03	32.5 ± 1.7	2.615 ± 0.083	

### Os Solution Reference Materials

The Os solution RMs are mainly used for monitoring the instrument status and inter-laboratory comparison of Os isotope ratio measurement results. The mostly used Os solution RMs include: JMC Os standard from University of Maryland (UMd) and DROsS from Durham University (or IAGeo, International Association of Geoanalysts). The other Os solution RMs mainly include: Os standard from Dept. Terrestrial Magmatism (DTM) and LOsST from Montanuniversität Leoben. These Os solution RMs have quite different ^187^Os/^188^Os ratios (Table 4).

**TABLE 4:** Os isotopic compositions for UMd, DROsS, DTM and LOsST Os solution reference materials.

RM	^186^Os/^188^Os	^187^Os/^188^Os	Reference
UMd (mean ± 2SD, n = 31)	0.1198458 ± 0.0000015	0.1137852 ± 0.0000023	Day et al. (2017)
DROsS (mean ± 2SD, n = 8)	0.119929 ± 0.000006	0.160924 ± 0.000004	Luguet et al. (2008)
DTM (mean ± 2SD, n = 14)	0.119965 ± 0.000004	0.173927 ± 0.000005	
LOsST (mean ± 2SD, n = 7)	0.119828 ± 0.000016	0.106917 ± 0.000015	Nowell et al. (2008a)

### Osmium Gravimetric Calibration Standards

Selby and Creaser (2001) and Markey et al. (2007) reported that the actual Os content of (NH_4_)_2_OsCl_6_ can be precisely determined by ignition under an atmosphere of 2% H_2_/98% N_2_. Nevertheless, the question still remains if it is accurate enough to calculate the stoichiometry. With this method, they produced an Os gravimetric standard for calibration of Os spike (usually ^190^Os).

## Concluding Remarks and Prospects

In the last two to three decades, a lot of progress has been made in the development of methods for Os isotope ratio and Re-PGE mass fraction measurements in geological samples. This progress mainly include the improvements of sample digestion, analyte/matrix separation, and mass spectrometric determination methods for bulk sample analyses, as well as the development of *in-situ* measurement techniques.

The developments of analytical methods allow for more accurate determinations of Os isotope ratios and Re-PGE mass fractions in various geological materials. In the current state-of-the-art Re-Os and PGE analysis, complete digestion of Re-PGE bearing minerals with low procedural blank, and selective dissolution of targeted Re-PGE bearing phases dependent on analytical purposes still remains challenging. In addition, the stoichiometry of the Os gravimetric calibration standard remains questionable. We also require further efforts in developing and providing well-characterized, matrix-matched RMs. In the future, in addition to instrumentation, the most important aspect for Os isotope ratio and Re-PGE mass fraction measurements is to further lower the laboratory blank levels of these elements or to increase sample amounts for digestion for low Os samples to improve the sample/blank ratios, such that the applications of the Re-Os and PGE systems in geosciences can be further extended. These may include more accurate LLHR sulfide dating of metal ore deposits, more accurate black shale Re-Os dating and Os isotope stratigraphy, and more accurate dating of petroleum system. In addition, complete or selective sample digestion dependent on analytical purposes, is also important for Re-Os geochronology and PGE geochemistry in the future.
